# Independent Influence of Blood Pressure on QTc Interval: Results from a General Chinese Population

**DOI:** 10.1155/2019/1656123

**Published:** 2019-07-08

**Authors:** Guo-Zhe Sun, Ying Zhou, Ning Ye, Shao-Jun Wu, Ying-Xian Sun

**Affiliations:** Department of Cardiovascular Medicine, The First Hospital of China Medical University, Shenyang, Liaoning 110001, China

## Abstract

**Aims:**

We performed the current study primarily to characterize the independent association of blood pressure with heart rate-corrected QT (QTc) interval after adjusting for cardiovascular confounding factors and left ventricular mass (LVM) in a large general population in China.

**Methods:**

All enrolled 10,553 permanent residents with age ≥ 35 years from Liaoning Province were investigated by a questionnaire and then subjected to physical examinations, laboratory analyses, and electrocardiogram (ECG) as well as echocardiogram at the same visit. Multivariate linear and logistic regression analyses were conducted to assess the independent association of blood pressure with QTc interval.

**Results:**

Hypertensive subjects had significantly longer QTc interval and higher prevalence of prolonged QTc interval compared with normotensive ones in all subgroups stratified by gender and left ventricular hypertrophy (LVH) (all* P*s ≤ 0.001). Multiple relevant clinical confounding factors and LVM were all adjusted in the multivariate linear and logistic regression analyses. As a result, both systolic blood pressure (SBP) and diastolic blood pressure (DBP) were independently associated with QTc interval (*β* = 0.12 and 0.16, respectively;* P*s < 0.001). Furthermore, as categorical variables, hypertension was independently associated with prolonged QTc interval (OR = 1.71;* P* < 0.001). Sex-specific analyses revealed that the independent associations were detected in both males and females (all* P*s < 0.001).

**Conclusions:**

These key findings of the current study highlighted the fact that hypertension was significantly associated with prolonged QTc interval and the correlations were independent of confounding factors and LVM.

## 1. Introduction

The prolongation of the heart rate-corrected QT (QTc) interval on electrocardiogram (ECG) may cause a number of adverse outcomes, including ventricular arrhythmias, sudden cardiac death, and all-cause mortality [1–4]. To help make population-based intervention strategies for this serious problem, great efforts have been made to identify the risk factors for QTc interval prolongation. Left ventricular hypertrophy (LVH), which can be assessed by echocardiography, has been considered as an independent risk factor for incident ventricular arrhythmias and sudden cardiac death [5, 6], possibly due to its influence on the prolonged QTc interval and increased QT dispersion [7, 8].

Elevated blood pressure is a well-known key factor for LVH. Previous population-based studies proposed a significant positive relationship between blood pressure and QTc interval [9, 10]. However, no data to date has showed whether the prolongation of QTc interval in hypertension is independent of the increase of left ventricular mass (LVM) or not. More interesting is that one recent research argued that prolonged QT interval is associated with blood pressure rather than LVM in spontaneously hypertensive rats [11]. Therefore, the present study was performed to explore the independent association of blood pressure with QTc interval and to determine whether this relationship depends on LVM in a large general population in China.

## 2. Materials and Methods

A multistage, random, stratified, cluster-sampling scheme was performed in this study. The details about research design, data collection, and measurements have been described previously [12]. This study was approved by the Ethics Committee of China Medical University, and all the procedures were performed in accordance with the ethical standards. Written consent was obtained from all participants or their proxies after they had been informed of the objectives, benefits, medical items, and confidentiality agreement of personal information. All of the eligible permanent residents aged ≥ 35 years from each village were invited to attend the study, with a response rate of 85.3% (11,956/14,016). Participants with pregnancy, malignant tumor, and severe mental disorders were excluded from the present study. Then a total of 1,403 subjects with missing data, poor echocardiogram or ECG quality, complete left or right bundle branch block, atrial fibrillation, or ventricular paced rhythm were excluded from the study, leaving 10,553 subjects for the final analyses (4,775 males and 5,778 females).

Weight and height were measured to the nearest 0.1 kg and 0.1 cm, respectively, with the participants in lightweight clothing without shoes. The body mass index (BMI) was calculated as weight in kilograms divided by the square of height in meters. Blood pressure was measured three times at two-minute intervals after at least five minutes of rest using a standardized automatic electronic sphygmomanometer (HEM-907; Omron, Kyoto, Japan) according to the American Heart Association. Two doctors calibrated the Omron device every month using a standard mercury sphygmomanometer according to the British Hypertension Society protocol [13]. The mean of three measurements was calculated and used in all analyses. In accordance with the JNC 7 Guidelines [14], hypertension was defined as a systolic blood pressure (SBP) ≥ 140 mmHg and/or a diastolic blood pressure (DBP) ≥ 90 mmHg and/or the use of antihypertensive medications.

Fasting blood samples were collected in the morning after at least 8 h of fasting for all participants. Blood samples were obtained from an antecubital vein using BD Vacutainer tubes containing EDTA (Becton, Dickinson and Co., Franklin Lakes, NJ, USA). Serum was subsequently isolated from whole blood, and all serum samples were frozen at -20°C for testing at a central, certified laboratory. Fasting plasma glucose (FBG), total cholesterol (TC), triglycerides (TG), high density lipid cholesterol (HDL-C), low density lipid cholesterol (LDL-C), serum uric acid (SUA), serum calcium, potassium and magnesium, and other routine blood biochemical indexes were analyzed on an autoanalyzer (Olympus AU640 Autoanalyzer; Olympus Corp., Kobe, Japan).

Twelve-lead resting, ten-second ECGs (25 mm/second paper speed and 10 mm/mV amplitude) were performed on all participants by well-trained cardiologists using an ECG machine (MAC 5500; GE Healthcare, Little Chalfont, Buckinghamshire, UK). Then, the digital ECGs were transferred into the MUSE Cardiology Information System (version 7.0.0; GE Healthcare) and analyzed automatically. The mean heart rate was read by the MUSE and used in the analyses. QTc intervals were calculated and recorded by the Bazett formula. Prolonged QTc interval was defined according to a scientific statement from the American Heart Association, which recommends the cut points of 460 milliseconds or longer in females and 450 milliseconds or longer in males [15].

Echocardiograms were obtained through a commercially available Doppler echocardiograph (Vivid; GE Healthcare) with a 3.0-MHz transducer. Echocardiogram analyses and readings were performed by three doctors specialized in echocardiography, and two other specialists were called in if questions or uncertainty arose. Measurements were performed according to the recommendations of the American Society of Echocardiography [16]. Left ventricular mass index (LVMI) was calculated according to body surface area. And LVH was defined as a LVMI > 115 g/m^2^ in males and > 95 g/m^2^ in females.

All statistical analyses were performed using SPSS 17.0 software (SPSS Inc., Chicago, IL, USA). Differences between groups were compared using a two-tailed Student's* t*-test for continuous variables and a *χ*^2^ test for categorical variables. The mean QTc interval and prevalence of prolonged QTc interval by hypertension and LVH were calculated and presented. Multivariate linear and logistic regression analyses were conducted for the independent associations of elevated blood pressure with QTc interval prolongation. Data were expressed as odds ratio (OR) and 95% confidence interval (CI), *β* and 95% CI, mean ± standard deviation, or percentage; a* P* < 0.05 was considered as statistically significant.

## 3. Results

Of the original 11,956 participants, 1,403 subjects were excluded from the analysis, leaving a total of 10,553 participants (4,775 males and 5,778 females) for the final analyses.  Table 1 presents the baseline characteristics of the study population according to QTc interval. The subjects with prolonged QTc interval were older with a lower percentage of men compared to those with normal QTc interval (*P*s < 0.001). Participants with prolonged QTc interval had significantly higher levels of BMI, SBP, DBP, FBG, TC, TG, LDL-C, SUA, calcium, LVMI, and heart rate and lower levels of potassium (all* P*s < 0.05). In addition, they had a lower percentage of alcohol drinkers and had higher proportions of heart disease history and self-reported medication use (all* P*s < 0.05). Furthermore, participants with prolonged QTc interval exhibited a higher prevalence of LVH than those with normal QTc interval (*P* < 0.001).

**Figure 1:**
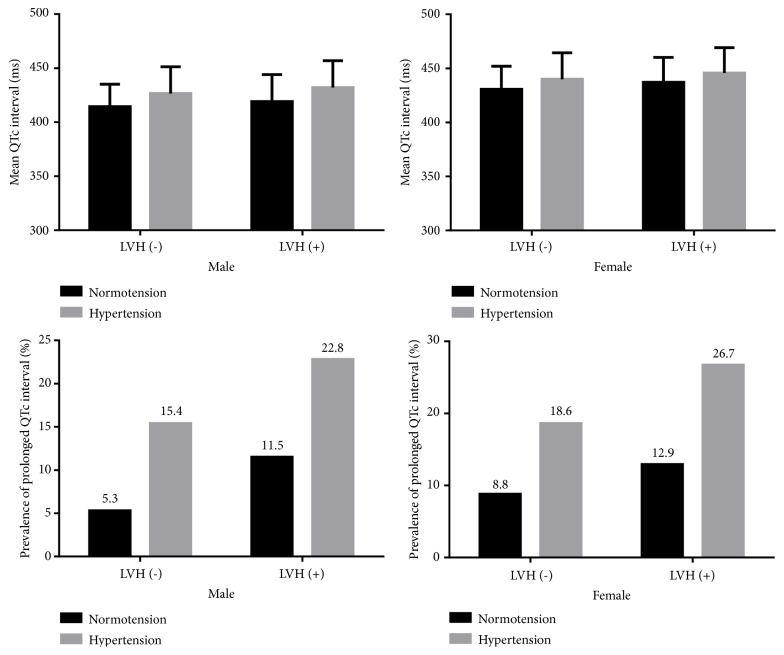
Gender-specific mean heart rate-corrected QT (QTc) interval and prevalence of prolonged QTc interval stratified by hypertension and left ventricular hypertrophy (LVH).

Figure 1 presents the sex-specific mean QTc interval and prevalence of prolonged QTc interval stratified by hypertension and LVH. As a result, among both males and females, hypertensive participants had significantly longer QTc interval compared with normotensive ones no matter whether they have LVH or not (all* P*s ≤ 0.001). Further, the prevalence of prolonged QTc interval in hypertensive subjects was significantly higher than those with normotension in all subgroups stratified by gender and LVH (all* P*s < 0.001).

Table 2 presents the multivariate linear regression analyses for the association between SBP, DBP, and QTc interval. Three models were adopted to explore the linear relationships. Among the total population, a significant correlation was identified between SBP, DBP, and QTc interval following adjustments for age, gender, race, BMI, FBG, TC, TG, LDL-C, HDL-C, SUA, current smoking and drinking status, serum calcium, potassium and magnesium, heart rate, history of heart disease, any medication used, and LVMI (*β* = 0.12 and 0.16 for SBP and DBP, respectively;* P*s < 0.001). Similar trends were found among both males and females (all* P*s < 0.001).

Table 3 presents the multivariate logistic regression analyses for the association between hypertension and QTc interval prolongation. After multivariate adjustments in model 3, hypertensive subjects also had a significantly increased risk for prolonged QTc interval compared to normotensive ones with OR of 1.71 (95% CI: 1.48–1.98;* P* < 0.001). Sex-specific analyses revealed that the independent association was detected in both males (OR = 2.03, 95% CI: 1.60–2.59;* P* < 0.001) and females (OR = 1.56, 95% CI: 1.30–1.88;* P* < 0.001).

## 4. Discussion

In the current large sample of general permanent residents in China, we firstly evaluated the effects of hypertension on prolonged QTc interval. As a result, the mean level of QTc interval increased with elevating blood pressure in both sexes, which was in line with previous studies [10, 17]. Hypertension was a pandemic clinical issue and our results identified a higher prevalence of prolonged QTc interval in hypertensive subjects no matter whether they have LVH or not. After adjusting for multiple clinical covariates, hypertension was found significantly and independently associated with prolonged QTc interval. Along with the adding of LVMI/LVH in the multivariate regression models, the results remained significant although the positive associations were attenuated. In addition, fairly slight decreases in *β* and OR caused by the adjusting of LVMI/LVH demonstrated that the main effects of blood pressure on QTc interval were not mediated by LVM.

Our findings were consistent with but not the same as previous ones. One study with large sample from Japan detected a positive relationship between blood pressure and QTc interval [9], but it was conducted only in 80-year-old subjects and the association was statistically significant only for SBP. Another study from China demonstrated the significant correlations of SBP and DBP with QTc interval in both sexes [10]. However, the sample size was quite limited and all study subjects were hypertensive patients. Furthermore, these studies drew their conclusions without proper regard to echocardiographic LVH, which could be caused by high level of blood pressure and often coexisted with hypertension. Considerable studies have proved that QT interval was positively associated with LVM and explored as a potential supplementary measure for the diagnosis of echocardiographic LVH [18, 19]. Thus, LVM should be considered during exploring the independent correlation. Therefore, a large sample of general residents older than 35 years with the data of clinical factors, ECG, and echocardiogram all collected at the same visit is the major strength of our study.

Previous studies speculated that one of the underlying mechanisms for the influence of blood pressure on QTc interval might be the chronic changes in the myocardium, such as increased LVM due to continuous high afterload [20, 21]. However, it is a controversial topic. The recent animal experiments showed that prolonged QT interval is associated with blood pressure rather than LVM in spontaneously hypertensive rats [11]. Accordingly, we additionally adjusted LVMI/LVH as well as multiple relevant clinical confounding factors in the final model of regression analyses. As a result, the associations between blood pressure and QTc interval were attenuated only a little and remained significant. Therefore, we summarized a novel viewpoint that the effect of blood pressure on QTc interval was not mainly dependent on the increase of LVM. In addition, autonomic nervous system might be another factor modulating QTc interval [22, 23]. Sympathetic activation could lead to QTc interval prolongation, whereas parasympathetic activation might decrease the QTc interval [24, 25]. It has also been proved that high sympathetic nervous activity usually existed in hypertension [26]. The individual autonomic activity and hemodynamic adaptation may be another potential mechanism linking QTc and blood pressure. Heart rate has been considered as a fine index reflecting the balance between the sympathetic and parasympathetic limbs of the autonomic nervous system [27]. For this reason, we also adjusted the heart rate in the multivariate regression analyses to alleviate its potential mediating effect and to verify the independent relationship between blood pressure and QTc interval.

However, some limitations exist in our study. First, this was a cross-sectional design without examining the longitudinal associations of hypertension with QTc interval. Second, all the enrolled participants were from the same province, so the results could not apply to all of China or other populations elsewhere. Third, only subjects ≥ 35 years of age were included in the current study. Fourth, since the digital ECGs were analyzed by the MUSE system automatically, there might be some systematic errors in measurement that could not be ruled out. In addition, only the Bazett formula was used for the calculation of QTc interval, which might bring some bias even though heart rate was adjusted.

## 5. Conclusions

In this study, subjects with hypertension had relatively longer QTc interval and higher prevalence of prolonged QTc interval. After multiple relevant clinical confounding factors and LVM were all adjusted, we found that hypertension was independently and significantly associated with prolonged QTc interval.

## Figures and Tables

**Table 1:** Characteristics of the study population.

Variable	Normal QTc	Prolonged QTc	*P *value
	(*n* = 9,182)	(*n* = 1,371)	
Age, years	53.2 ± 10.3	56.8 ± 10.8	< 0.001
Male	4,239 (46.2)	536 (39.1)	< 0.001
Race of Han	8,708 (94.8)	1,303 (95.0)	0.751
BMI, kg/m^2^	24.7 ± 3.6	25.3 ± 3.9	< 0.001
SBP, mmHg	139.9 ± 22.5	152.6 ± 25.5	< 0.001
DBP, mmHg	81.3 ± 11.3	86.5 ± 13.0	< 0.001
Hypertension	4,359 (47.5)	982 (71.6)	< 0.001
FBG, mmol/L	5.84 ± 1.50	6.31 ± 2.27	< 0.001
TC, mmol/L	5.21 ± 1.07	5.40 ± 1.16	< 0.001
TG, mmol/L	1.59 ± 1.39	1.97 ± 1.96	< 0.001
LDL-C, mmol/L	2.90 ± 0.81	3.05 ± 0.85	< 0.001
HDL-C, mmol/L	1.41 ± 0.38	1.40 ± 0.40	0.441
SUA, mg/dL	4.87 ± 1.40	4.99 ± 1.53	0.007
Calcium, mmol/L	2.32 ± 0.13	2.34 ± 0.12	< 0.001
Potassium, mmol/L	4.21 ± 0.34	4.09 ± 0.38	< 0.001
Magnesium, mmol/L	0.85 ± 0.11	0.85 ± 0.08	0.117
Current smoker	3,243 (35.3)	474 (34.6)	0.590
Current drinker	2,073 (22.6)	264 (19.3)	0.006
LVIDd, cm	4.70 ± 0.41	4.74 ± 0.45	0.001
IVS, cm	0.87 ± 0.12	0.91 ± 0.14	< 0.001
PWT, cm	0.85 ± 0.10	0.88 ± 0.12	< 0.001
LVMI, g/m^2^	81.1 ± 18.0	86.5 ± 21.7	< 0.001
LVH	837 (9.1)	251 (18.3)	< 0.001
Heart rate, bpm	70.3 ± 11.6	79.9 ± 13.1	< 0.001
QTc interval, ms	423.3 ± 19.2	469.5 ± 18.9	< 0.001
History of heart disease†	830 (9.0)	210 (15.3)	< 0.001
Medication used‡	4,745 (51.7)	870 (63.5)	< 0.001

Abbreviations. BMI: body mass index; DBP: diastolic blood pressure; FBG: fasting blood glucose; HDL-C: high density lipid cholesterol; IVS: interventricular septum; LDL-C: low density lipid cholesterol; LVH: left ventricular hypertrophy; LVIDd: left ventricular internal diameter at end-diastole; LVMI: left ventricular mass index; PWT: posterior wall thickness; QTc: QT interval corrected for heart rate with Bazett's formula; SBP: systolic blood pressure; TC: total cholesterol; TG: triglycerides.

Note. Data are presented as mean ± standard deviation or *n *(%).

†Including coronary heart disease, arrhythmia, and heart failure.

‡Indicating any self-reported medication used in the past 2 weeks.

**Table 2:** Multivariate linear regression for association between blood pressure and QTc interval (ms).

Group	Model 1		Model 2		Model 3	
	*β* (95% CI)	*P *value	*β* (95% CI)	*P *value	*β* (95% CI)	*P *value
Total						
SBP, mmHg	0.23 (0.21–0.25)	< 0.001	0.14 (0.12–0.16)	< 0.001	0.12 (0.10–0.14)	< 0.001
DBP, mmHg	0.46 (0.42–0.50)	< 0.001	0.20 (0.16–0.24)	< 0.001	0.16 (0.12–0.20)	< 0.001
Male						
SBP, mmHg	0.28 (0.25–0.31)	< 0.001	0.19 (0.16–0.21)	< 0.001	0.16 (0.13–0.19)	< 0.001
DBP, mmHg	0.56 (0.51–0.62)	< 0.001	0.25 (0.20–0.30)	< 0.001	0.20 (0.14–0.25)	< 0.001
Female						
SBP, mmHg	0.19 (0.16–0.22)	< 0.001	0.11 (0.09–0.14)	< 0.001	0.09 (0.07–0.12)	< 0.001
DBP, mmHg	0.37 (0.32–0.42)	< 0.001	0.15 (0.10–0.20)	< 0.001	0.12 (0.07–0.17)	< 0.001

Abbreviations as in Table 1.

Note. Model 1: adjusted for age, gender, race, SBP, and DBP; Model 2: additional adjustments for BMI, FBG, TC, TG, LDL-C, HDL-C, SUA, current smoking, drinking status, serum calcium, potassium and magnesium, heart rate, history of heart disease, and any medication used; Model 3: additional adjustments for LVMI.

**Table 3:** Logistic regression analyses for association between hypertension and prolonged QTc interval.

Group	Prolonged QTc	Model 1		Model 2		Model 3	
	*n* (%)	OR (95% CI)	*P *value	OR (95% CI)	*P *value	OR (95% CI)	*P *value
Total							
Normotension	389 (7.5)	1		1		1	
Hypertension	982 (18.4)	2.48 (2.17–2.82)	< 0.001	1.79 (1.55–2.07)	< 0.001	1.71 (1.48–1.98)	< 0.001
Male							
Normotension	120 (5.4)	1		1		1	
Hypertension	416 (16.3)	2.99 (2.40–3.72)	< 0.001	2.14 (1.69–2.72)	< 0.001	2.03 (1.60–2.59)	< 0.001
Female							
Normotension	269 (9.0)	1		1		1	
Hypertension	566 (20.3)	2.23 (1.89–2.63)	< 0.001	1.63 (1.36–1.95)	< 0.001	1.56 (1.30–1.88)	< 0.001

Abbreviations. CI: confidence interval; OR: odds ratio; QTc: QT interval corrected for heart rate with Bazett's formula.

Note. Model 1: adjusted for age, gender, and race; Model 2: additional adjustments for BMI, FBG, TC, TG, LDL-C, HDL-C, SUA, current smoking, drinking status, serum calcium, potassium and magnesium, heart rate, history of heart disease, and any medication used; Model 3: additional adjustments for LVH.

## Data Availability

The data used to support the findings of this study are available from the corresponding author upon request.

## References

[1] Hobbs J. B., Peterson D. R., Moss A. J. (2006). Risk of aborted cardiac arrest or sudden cardiac death during adolescence in the long-QT syndrome. *Journal of the American Medical Association*.

[2] Algra A., Tijssen J. G. P., Roelandt J. R. T. C., Pool J., Lubsen J. (1991). QTc prolongation measured by standard 12-lead electrocardiography is an independent risk factor for sudden death due to cardiac arrest. *Circulation*.

[3] Straus S. M., Kors J. A., de Bruin M. L. (2006). Prolonged QTc interval and risk of sudden cardiac death in a population of older adults. *Journal of the American College of Cardiology*.

[4] Okin P. M., Devereux R. B., Howard B. V., Fabsitz R. R., Lee E. T., Welty T. K. (2000). Assessment of QT interval and QT dispersion for prediction of all-cause and cardiovascular mortality in American Indians: the strong heart study. *Circulation*.

[5] Bluemke D. A., Kronmal R. A., Lima J. A. (2008). The relationship of left ventricular mass and geometry to incident cardiovascular events. *Journal of the American College of Cardiology*.

[6] Stewart M. H., Lavie C. J., Shah S. (2018). Prognostic implications of left ventricular hypertrophy. *Progress in Cardiovascular Diseases*.

[7] Dimopoulos S., Nicosia F., Donati P. (2008). QT dispersion and left ventricular hypertrophy in elderly hypertensive and normotensive patients. *Angiology*.

[8] Oikarinen L., Nieminen M. S., Viitasalo M. (2001). Relation of QT interval and QT dispersion to echocardiographic left ventricular hypertrophy and geometric pattern in hypertensive patients. The LIFE study.. *Journal of Hypertension*.

[9] Matsumura K., Takata Y., Ansai T. (2004). Association of QT interval with blood pressure in 80-year-old subjects. *Hypertension Research*.

[10] Peng S., Yu Y., Hao K. (2006). Heart rate–corrected QT interval duration is significantly associated with blood pressure in Chinese hypertensives. *Journal of Electrocardiology*.

[11] Klimas J., Stankovicova T., Kyselovic J., Bacharova L. (2009). Prolonged QT interval is associated with blood pressure rather than left ventricular mass in spontaneously hypertensive rats. *Clinical and Experimental Hypertension*.

[12] Sun G., Ye N., Chen Y., Zhou Y., Li Z., Sun Y. (2017). Early repolarization pattern in the general population: prevalence and associated factors. *International Journal of Cardiology*.

[13] O'Brien E., Petrie J., Littler W. (1990). The British Hypertension Society protocol for the evaluation of automated and semi-automated blood pressure measuring devices with special reference to ambulatory systems. *Journal of Hypertension*.

[14] Chobanian A. V., Bakris G. L., Black H. R. (2003). The seventh report of the joint national committee on prevention, detection, evaluation, and treatment of high blood pressure: the JNC 7 report. *The Journal of the American Medical Association*.

[15] Rautaharju P. M., Surawicz B., Gettes L. S. (2009). AHA/ACCF/HRS recommendations for the standardization and interpretation of the electrocardiogram: part IV: the ST segment, T and U waves, and the QT interval: a scientific statement from the american heart association electrocardiography and arrhythmias committee, council on clinical cardiology; the american college of cardiology foundation; and the heart rhythm society. endorsed by the international society for computerized electrocardiology. *Journal of the American College of Cardiology*.

[16] Lang R. M., Bierig M., Devereux R. B. (2005). Recommendations for chamber quantification: a report from the American Society of Echocardiography's guidelines and standards committee and the Chamber Quantification Writing Group, developed in conjunction with the European Association of Echocardiography, a branch of the European Society of Cardiology. *Journal of the American Society of Echocardiography*.

[17] Mozos I., Serban C. (2011). The relation between QT interval and T-wave variables in hypertensive patients. *Journal of Pharmacy and Bioallied Sciences*.

[18] Chapman N. (2001). QT intervals and QT dispersion as measures of left ventricular hypertrophy in an unselected hypertensive population. *American Journal of Hypertension*.

[19] Salles G., Leocadio S., Bloch K., Nogueira A. R., Muxfeldt (2005). Combined QT interval and voltage criteria improve left ventricular hypertrophy detection in resistant hypertension. *Hypertension*.

[20] Oikarinen L., Nieminen M. S., Toivonen L. (2003). Relation of QT interval and QT dispersion to regression of echocardiographic and electrocardiographic left ventricular hypertrophy in hypertensive patients: the Losartan Intervention For Endpoint Reduction (LIFE) study. *American Heart Journal*.

[21] Baillard C., Mansier P., Ennezat P. V. (2000). Converting enzyme inhibition normalizes QT interval in spontaneously hypertensive rats. *Hypertension*.

[22] Abildskov J. A. (1976). Adrenergic effects on the QT interval of the electrocardiogram. *American Heart Journal*.

[23] Browne K. F., Zipes D. P., Heger J. J., Prystowsky E. N. (1982). Influence of the autonomic nervous system on the Q-T interval in man. *American Journal of Cardiology*.

[24] Annila P., Yli-Hankala A., Lindgren L. (1993). Effect of atropine on the qt interval and t-wave amplitude in healthy volunteers. *British Journal of Anaesthesia*.

[25] Diedrich A., Jordan J., Shannon J. R., Robertson D., Biaggioni I. (2002). Modulation of QT interval during autonomic nervous system blockade in humans. *Circulation*.

[26] Marfella R., Gualdiero P., Siniscalchi M. (2003). Morning blood pressure peak, QT intervals, and sympathetic activity in hypertensive patients. *Hypertension*.

[27] Grande D., Iacoviello M., Aspromonte N. (2018). The effects of heart rate control in chronic heart failure with reduced ejection fraction. *Heart Failure Reviews*.

